# Levels and Predictors of Anxiety and Depression in Turkish Pregnant Woman During the Covid-19 Pandemic

**DOI:** 10.1055/s-0041-1741033

**Published:** 2022-02-25

**Authors:** Yılda Arzu Aba, Ozlem Dulger, Bulat Aytek Sık, Ozan Ozolcay

**Affiliations:** 1 Department of Nursing, Faculty of Health Sciences, Bandırma Onyedi Eylül University, Balıkesir, Turkey; 2 Deparment of Gynecology and Obstetrics, Süleymaniye Education and Research Hospital, Istanbul, Turkey; 3 Department of Reproductive Endocrinology and Infertility, Istanbul Aydın University, Istanbul, Turkey; 4 Karamanoğlu Mehmetbey University, School of Medicine, Department of Gynecology and Obstetrics, Karaman, Turkey

**Keywords:** Covid-19, anxiety, depression, pregnant women, Covid-19, ansiedade, depressão, mulheres grávidas

## Abstract

**Objective** In addition to being a medical phenomenon, pandemics affect the individual and society on several levels and lead to disruptions. In the pandemic process, different groups in the population, including pregnant women as a defenseless group, are subjected to psychological threat. The present study aimed to determine the levels of anxiety and depression and related factors in pregnant women during the the coronavirus disease 2019 (Covid-19) pandemic.

**Methods** The present cross-sectional study was conducted with 269 pregnant women through face-to-face interviews held in Istanbul, Turkey. Regarding the data collection tools, the Cronbach α reliability coefficient was of 0.90 for the Beck Anxiety Inventory, and of 0.85 for the Beck Depression Inventory.

**Results** Among the participating pregnant women, 30.5% had mild, 17.5% had moderate, and 5.9% had severe anxiety symptoms, whereas 35.3% had mild, 16.7% had moderate, and 2.2% had severe depression symptoms. We found that those who were concerned about their health had 5.36 times ( *p* = 0.04) more risk of developing anxiety, and 4.82 times ( *p* = 0.01) more risk of developing depression than those who were not concerned. Those who had a history of psychiatric disease had 3.92 times ( *p* = 0.02) more risk of developing anxiety than those without it.

**Conclusion** We determined that about half of the pregnant women included in the study had some degree of anxiety and depression during the COVID-19 pandemic. The risk factors for anxiety and depression among the pregnant women were determined as smoking, concerns about health and getting infected with the coronavirus, history of psychiatric disease, and undergoing regular antenatal care.

## Resumo

**Objetivo** Além de ser um fenômeno médico, as pandemias também afetam o indivíduo e a sociedade em vários níveis, e causam perturbações. No processo de pandemia, diferentes grupos da população, incluindo mulheres grávidas como um grupo indefeso, estão sujeitos a ameaças psicológicas. Este estudo teve como objetivo determinar os níveis de ansiedade e depressão e os fatores relacionados em mulheres grávidas durante a pandemia de doença do coronavírus 2019 ( *coronavirus disease* 2019, Covid-19, em inglês).

**Métodos** Este estudo transversal foi realizado com 269 mulheres grávidas por meio de entrevistas pessoais em Istambul, Turquia. Com relação às ferramentas de coleta de dados, o coeficiente de confiabilidade alfa de Cronbach foi de 0,90 para a Escala de Ansiedade de Beck, e de 0,85 para a Escala de Depressão de Beck.

**Resultados** Entre as gestantes participantes, 30,5% apresentaram sintomas de ansiedade leves, 17,5%, moderados, e 5,9%, graves, ao passo que 35,3% apresentaram sintomas de depressão leves, 16,7%, moderados, e 2,2%, graves. Verificou-se que as participantes que se preocupavam com sua saúde tinham 5,36 vezes ( *p* = 0,04) mais risco de desenvolver ansiedade e 4,82 vezes ( *p* = 0,01) mais risco de desenvolver depressão do que aquelas que não se preocupavam. As pacientes que tinham histórico de doença psiquiátrica tinham 3,92 vezes ( *p* = 0,02) mais risco de desenvolver ansiedade do que as que não tinham.

**Conclusão** Determinou-se que cerca de metade das gestantes incluídas no estudo tiveram algum nível de ansiedade e depressão durante a pandemia de Covid-19. Os fatores de risco para ansiedade e depressão nas gestantes foram determinados como tabagismo, preocupação com a saúde e infecção pelo coronavírus, histórico de doença psiquiátrica, e cuidados pré-natais regulares.

## Introduction

After reporting cases of pneumonia of unknown etiology in the city of Wuhan, Hubei province, China, on December 31st, 2019, on January 7th, 2020, the World Health Organization (WHO) declared the discovery of a novel coronavirus (2019-nCoV) that had not been determined in humans before. The virus, which causes a condition called coronavirus disease 2019 (Covid-19), then spread to the entire world rapidly, in what constituted a pandemic. 1 Although the virus was observed for the first time in China, 2 countries in Europe 3 and the United States 4 have been the most affected since, and the first case in Turkey was reported on March 11th, 2020. By August 9th, 2021, the total number of cases reported in Turkey was 5,895,841, while the number of deaths was 52,088. 5

In addition to being a medical phenomenon, pandemics also affect the individual and society on several levels, and lead to disruptions. This is because people who experience panic and stress as the perception of the threat posed by the contagious disease increases display different behaviors than at other times. The way in which individuals and society manage and cope with the emotional and psychosocial effects of the uncertainty and crisis that emerge during the pandemic periods is important. 6 It is accepted as natural for people to display behaviors of protection and avoidance with the feelings of fear and panic while facing an unpredictable situation like an epidemic disease. The existing situation leads to health problems such as stress, anxiety, depressive symptoms, insomnia, denial, anger, and fear. 7 Pandemic-related questions without precise answers, such as when it will end and treatment/protection methods, and the constant exposure to an information flow regarding the pandemic and its effects and recommendations/bans, like social isolation and staying home as much as possible, may especially affect the mental health of the society in a negative way. It has been reported 8 that signs like anxiety, depression, fear, stress and sleep disorders are being observed more frequently during the Covid-19 pandemic in healthy individuals. At the same time, these symptoms are also common among Covid-19 patients. 9 In the pandemic process, different groups in the population, including pregnant women as a defenseless group, are subjected to psychological threat. In addition to the unknown nature of the virus, the lack of information about its transmission, reproduction, risk factors, mortality rates and maternal and fetal effects may pose a risk not only for the physical health but also for the mental health of individuals. 10

While pregnancy is one of the most special situations that can be experienced by a woman throughout her life, transition to motherhood is a complex process in which physiological and psychological changes, as well as changes in sociality, take place, and several psychosocial factors are in interaction with each other. 11 12 13 There are many factors that disrupt the mental health of the mother during pregnancy. These may include a history of previous depression, family history of depression, unwanted pregnancies, history of miscarriage and curettage, history of stillbirth, low socioeconomic status, anxiety related to the fetus, parenthood stress, and negative life experiences. 14 15 16 17 Mental health disorders are a cause of morbidity that is prevalently seen during pregnancy, and studies 18 have reported depression in 12% and anxiety disorders in 22% of pregnant women, especially in the second and third trimesters. Moreover, studies conducted in Canada 19 and Turkey 20 have revealed that pregnant women had higher levels of stress, anxiety and depression during the COVID-19 pandemic in comparison to before the pandemic. A study conducted among Iranian pregnant women by Effati-Daryani et al. 21 showed anxiety symptoms in 43.9% of the sample, and depression symptoms in 32.7%, at degrees varying from mild to highly severe. Consequently, considering the effects of mental disorders on maternal and fetal health, in order to determine and improve the psychological state of pregnant women and prevent complications, the most important responsibility of healthcare professionals is to provide psychological support and help in the recovery process. Therefore, the present study aimed to determine the levels of anxiety and depression and their related factors in pregnant women during the COVID-19 pandemic.

## Methods

### Design and Participants

The present is a cross-sectional study conducted to determine the levels of anxiety and depression levels of pregnant women and their associated factors during the COVID-19 pandemic.

The population of the study consisted of all pregnant women who visited the pregnancy polyclinics of three hospitals in the province of Istanbul, Turkey, between July 15th and September 15th, 2020 and volunteered to participate in the study. With a Type-I error rate of 0.05, ratios of 50% for the H _o_ hypothesis and 60% for the H _1_ hypothesis, and a testing power of 99%, the minimum sample size needed for the study was calculated as 269 individuals.

### Inclusion Criteria

Based on the objectives of the present study and a literature review, the inclusion criteria were as follows:

- Being in the first, second or third trimesters of pregnancy; and
- Being able to read and write in Turkish.

### Exclusion Criteria

- Experiencing pregnancy complications;
- Getting diagnosed with Covid-19; and
- Being under psychiatric treatment.

### Data Collection Tools

As the data collection instruments, we used a personal information form developed to determine the descriptive characteristics of the pregnant women and their risk factors that could be associated with anxiety and depression, the Beck Anxiety Inventory (BAI) to assess their anxiety symptoms, and the Beck Depression Inventory (BDI) to assess their depression symptoms.

**Personal information form:** Developed by the researchers and including twenty questions, this form asked for information on some sociodemographic characteristics of the pregnant women, their general health status and habits, obstetric characteristics, and psychosocial factors during the pandemic.

**Beck Anxiety Inventory:** Developed by Beck et al. (1988), 22 it measures the frequency of the anxiety symptoms experienced by the individual. Its validity and reliability study in Turkey was performed by Ulusoy et al. (1998). 23 It is a 4-point, 21-item Likert-type self-assessment scale in which each item is scored between 0 and 3. By adding the scores of all 21 items, scores in the range of 0 to 63 are obtained, and higher scores indicate higher levels of anxiety symptoms. The results of the BAI are: as 0 to 7 points – no anxiety symptoms; 8 to 15 points – mild anxiety; 16 to 25 points – moderate anxiety; and 25 to 63 points – severe anxiety. In the validity and reliability study by Ulusoy et al., 23 the cutoff point of the scale was determined as 18. In the present study, the Cronbach alpha reliability coefficient of the BAI was determined as 0.90.

**Beck Depression Inventory:** Developed by Beck et al. (1961) 24 and adapted into the Turkish language by Hisli (1989), 25 the BDI consists of 21 items on depressive symptoms such as pessimism, sense of failure, self-dissatisfaction, guilt, irritability, fatigability, loss of appetite, indecisiveness, insomnia and social withdrawal. Each item contains 4 self-assessment statements and a 4-point Likert-type scoring system with scores ranging from 0 to 3 based on the severity of the depression. By adding the scores of all 21 items, scores in the range of 0 to 63 are obtained, and higher scores indicate higher levels of depressive symptoms. In the validity and reliability study conducted by Hisli, 25 the cut-off score of the scale was determined as 17. The results of the BDI are: 0 to 9 points – no depressive symptoms; 10 to 16 points – mild depressive symptoms; 17 to 29 points – moderate depressive symptoms; and 30 to 63 points – severe depressive symptoms. 25 26 In the present study, the Cronbach alpha reliability coefficient of the BDI was determined as 0.85.

### Data Collection

The participants were informed verbally and in writing by the researchers about the objective and significance of the study. The pregnant women who agreed to participate and provided their written consent were directed to a private room where they would answer the forms in line with the rules enacted due to the pandemic. During data collection, it took each participant ∼ 10 to 15 minutes to fill out the forms.

### Ethical Considerations

To assess the ethical suitability of the study, an application was made to the Health Sciences Ethics Board at Bandırma Onyedi Eylül University, and approval was obtained under number 2020/264. Additionally, written institutional permissions were received from the hospitals in order to conduct the study.

### Data Analysis

The data were analyzed by using the Statistical Package for the Social Sciences (IBM SPSS Statistics for Windows, IBM Corp., Armonk, NY, US) software, version23.0. The Kolmogorov-Smirnov test was used to examine if the variables conformed with a normal distribution. To examine the main effects of the sociodemographic data on the dependent variables of anxiety and depression, multivariate analysis of variance (MANOVA) was performed. Binary logistic regression analysis was used to determine the risk factors for anxiety and depression. The quantitative data are presented as mean and standard deviation values. The results were interpreted in a 95% confidence interval and a significance level of *p* < 0.05.

## Results

The ages of the pregnant women in the sample varied from 20 to 45 years, and their mean age was of 31.74 ± 5.04 years. The mean gestational week was 24.50 ± 8.77, and 62.8% ( *n* = 169) did not have any living children. The mean length of time they stayed home during the pandemic was of 56.93 ± 9.65 days ( Table 1 ).

**Table 1:** Descriptive Characteristics of the Participants and Mean BAI and BDI Scores

Characteristics ( *n* = 269)	Mean ± SD		
Age (years)	31.74 ± 5.044		
Week of pregnancy	24.50 ± 8.77		
Stayed at home during the pandemic (days)	56.93 ± 9.65		
Level of schooling	n (%)	BAI: mean ± SD	BDI: mean ± SD
Primary school	21 (7.8)	10.2 ± 5.9	10.7 ± 6.7
High school	57 (21.2)	10.8 ± 9	10.5 ± 6.1
Bachelor's degree	191 (71)	10.7 ± 8.9	10.4 ± 7.2
Economic status			
Income lower than expenses	14 (5.2)	11.4 ± 5.9	12.1 ± 6.6
Income equal to expenses	214 (79.6)	10.6 ± 8.7	10.5 ± 7
Income higher than expenses	41 (15.2)	10.9 ± 9.6	9.3 ± 6.4
Occupation			
Employed	176 (65.4)	10.9 ± 8.8	10.1 ± 6.6
Unemployed	93 (34.6)	10.1 ± 8.5	11 ± 7.5
Smoking			
Yes	20 (7.4)	14.3 ± 9.1	14.4 ± 7.9
No	249 (92.6)	10.4 ± 8.6	10.1 ± 6.7
Regular physical activity			
Yes	97 (36.1)	10.4 ± 8.4	10.8 ± 6.6
No	172 (63.9)	10.8 ± 8.9	10.2 ± 7.1
Presence of social support			
Yes	203 (75.5)	10.5 ± 8.4	10.4 ± 6.9
No	66 (24.5)	11 ± 9.6	10.5 ± 7.1
Concerned about health			
Yes	188 (69.9)	12.7 ± 9.2	12 ± 7
No	81 (30.1)	5.9 ± 4.5	6.8 ± 5.3
Planned pregnancy			
Yes	222 (82.5)	9.9 ± 8.2	9.9 ± 6.9
No	47 (17.5)	14.1 ± 10	13 ± 6.2
Chronic disease			
Yes	20	11.1 ± 7	11.4 ± 6.3
No	249	10.6 ± 8.8	10.3 ± 7
History of psychiatric disease			
Yes	27 (10)	16.1 ± 8.7	15.5 ± 7.4
No	242 (90)	10 ± 8.5	9.8 ± 6.6
Concerned about infection by the coronavirus			
Yes	195 (72.5)	12.3 ± 9.2	11.2 ± 6.9
No	74 (27.5)	6.2 ± 5	8.3 ± 6.4
Measures taken during the pandemic			
Use of face mask	201 (74.7)	10.7 ± 9	10.5 ± 7.3
Use of face mask + gloves	49 (18.2)	9.7 ± 7.1	9.7 ± 5.9
Use of face mask + gloves + disinfectant	19 (7.1)	12.1 ± 8.9	11.2 ± 5.4
Following Covid-19 news			
Yes	254 (94.4)	10.6 ± 8.7	10.4 ± 7
No	15 (5.6)	12 ± 8.8	9.9 ± 5
Presence at home of an individual older than 65 years of age			
Yes	58 (21.6)	13.3 ± 8.5	10.5 ± 6.7
No	211 (78.4)	9.9 ± 8.6	10.4 ± 7
Receiving regular antenatal care			
Yes	222 (82.5)	9.5 ± 8	9.9 ± 6.6
No	7 (2.6)	15.4 ± 9.1	15.3 ± 8.4
I chose to delay	40 (14.9)	16.2 ± 10.2	12.2 ± 8

Abbreviations: Covid-19, coronavirus disease 2019; BAI, Beck Anxiety Inventory; BDI, Beck Depression Inventory; SD, standard deviation.

We determined that, while 46.1% ( *n* = 124) of the sample did not have anxiety, 30.5% ( *n* = 82) had mild, 17.5% ( *n* = 47) had moderate, and 5.9% ( *n* = 16) had severe levels of anxiety symptoms. We observed that, while 45.7% ( *n* = 123) of the participants did not have depressive symptoms, 35.3% ( *n* = 95) had mild, 16.7% ( *n* = 45) had moderate, and 2.2% ( *n* = 6) had severe levels of depressive symptoms ( Table 2 ).

**Table 2:** MANOVA Results of the BAI and BDI Scores Based on The Descriptive Characteristics of the Sample

Characteristics ( *n* = 269)	Scales	SS	df	MS	F	*p* -value	*η* ^2^
Level of schooling	BAI	29.31	2	14.65	0.25	0.78	0.00
	BDI	3.34	2	1.67	0.04	0.96	0.00
Economic status	BAI	36.50	2	18.25	0.32	0.73	0.00
	BDI	28.73	2	14.37	0.36	0.70	0.00
Employment status	BAI	11.63	1	11.63	0.20	0.65	0.00
	BDI	95.43	1	95.49	2.41	0.12	0.01
Smoking status	BAI	160.51	1	160.51	2.77	0.10	0.01
	BDI	174.04	1	174.04	4.39	0.03	0.01
Regular exercise	BAI	10.91	1	10.91	0.19	0.67	0.00
	BDI	16.11	1	16.11	0.41	0.52	0.00
Presence of social support	BAI	14.01	1	14.01	0.24	0.62	0.00
	BDI	4.62	1	4.62	0.12	0.73	0.00
Concern about health	BAI	794.87	1	794.87	13.73	< 0.00	0.05
	BDI	982.78	1	982.78	24.82	< 0.00	0.09
Planned pregnancy	BAI	306.20	1	306.20	5.29	0.02	0.02
	BDI	150.39	1	150.39	3.80	0.05	0.02
Chronic disease during pregnancy	BAI	28.79	1	28.79	0.50	0.48	0.00
	BDI	3.92	1	3.92	0.09	0.75	0.00
History of psychiatric disease	BAI	379.10	1	379.10	6.55	0.01	0.03
	BDI	441.02	1	441.02	11.14	0.00	0.04
Precautions taken during the pandemic	BAI	4.76	2	2.38	0.04	0.96	0.00
	BDI	4.25	2	2.18	0.05	0.95	0.00
Concerns of getting infected by the coronavirus	BAI	521.51	1	521.51	9.01	0.00	0.04
	BDI	17.98	1	17.98	0.45	0.50	0.00
Following Covid-19 news	BAI	30.59	1	30.59	0.53	0.46	0.00
	BDI	0.01	1	0.01	0.00	0.98	0.00
Presence at home of an individual older than 65 years of age	BAI	319.87	1	319.87	5.52	0.02	0.02
	BDI	16.12	1	16.12	0.41	0.52	0.00
Receiving regular antenatal care	BAI	813.50	2	406.75	7.03	0.00	0.05
	BDI	56.25	2	28.13	0.71	0.49	0.01

Abbreviations: *η* ^2^ , partial eta-squared; Covid-19, coronavirus disease 2019; BAI, Beck Anxiety Inventory; BDI, Beck Depression Inventory; MANOVA, multivariate analysis of variance; MS, mean squares; SD, standard deviation; SS, sum of squares.

Table 2 presents the comparison of the BAI and BDI scores based on the descriptive characteristics of the sample. Significant differences were found in the mean BAI scores in terms of concerns about health, status of planning the pregnancy, history of psychiatric disease, concerns of being infected with the coronavirus, presence of an individual older than 65 years of age at their residence, and regular follow-up status ( *p* < 0.05). While the mean BAI score of the pregnant women who were concerned about health was of 12.7, the mean score of those who were not concerned was of 5.9 ( *p* < 0.00). The mean BAI score of those with a planned pregnancy was of 9.9, and for those with an unplanned pregnancy, it was was 14.1 ( *p* = 0.02). Regarding history of psychiatric disease, the mean BAI score was of 16.1 among those who had it, while that of those without such a history was of 10 ( *p* = 0.01). Those who were concerned about getting infected with the coronavirus had a mean score of 12.3 on the BAI, whereas that of those who were not concerned was of 6.2 ( *p* = 0.00). The mean BAI score of the participants who shared their residence with individuals over the age of 65 was of 13.3, while for those that did not, it was of 9.9 ( *p* = 0.02). The mean BAI score of those who were continuing regular antenatal follow-ups was pf 9.5, while the mean score of those who delayed their follow-ups was of 16.2 ( *p* = 0.00).

In the present study, there were significant differences in the BDI scores regarding the participants' smoking status, concerns about health, and history of psychiatric disease ( *p* < 0.05). Among the participants, while the mean BDI score of those who were smoking was 14.4, the mean score of those who were not smoking was 10.1 ( *p* = 0.03). The mean BDI score of those who were concerned about health was 12, and for those who were not concerned, it was 6.8. And the mean BDI score of those with a history of psychiatric disease was 15.5, and for those without such a history, 9.8 ( *p* = 0.00).

Table 3 presents the logistic regression results for the risk factors affecting the anxiety and depression levels of the study participants. We found that those who were concerned about their health had 5.362 times ( *p* = 0.04) more risk iof developing anxiety, and 4.818 times ( *p* = 0.01)more risk of having depression than those who were not concerned. There was a significant difference between the participants whose pregnancies were planned and those whose pregnancies were unplanned in terms of the presence of anxiety ( *p* = 0.05). Those who had a history of psychiatric disease had 3.924 times ( *p* = 0.02) more risk of developing anxiety than those without such a history. There was a significant difference between the participants who delayed their regular antenatal follow-ups and those who continued their follow-ups in terms of the presence of anxiety ( *p* = 0.01).

**Table 3:** Risk factors affecting Anxiety and Depression levels

Characteristics	Beck Anxiety Inventory		Beck Depression Inventory	
	OR (95%CI)*	*p* -value	OR (95%CI)**	*p* -value
**Education level**				
Primary school	0.212 (0.015–3.004)	0.25	0.215 (0.027–1.718)	0.15
High school	1.494 (0.551–4.053)	0.43	0.93 (0.356–2.43)	0.88
**Income status**				
Income lower than expenses	0.147 (0.009–2.284)	0.17	2.441 (0.312–19.111)	0.40
Income equal to expenses	0.485 (0.172–1.368)	0.17	1.471 (0.477–4.537)	0.50
Employment status (no)	0.994 (0.406–2.436)	0.99	0.465 (0.208–1.038)	0.06
Smoking (no)	3.121 (0.824–11.818)	0.09	2.706 (0.826–8.861)	0.10
Regular exercise (no)	0.826 (0.354–1.928)	0.66	0.947 (0.432–2.076)	0.89
Presence of social support (no)	1.026 (0.385–2.731)	0.96	1.094 (0.443–2.699)	0.85
Concern about health (no)	5.362 (1.092–26.33)	0.04	4.818 (1.451–15.999)	0.01
Planned pregnancy (no)	0.366 (0.134–0.996)	0.05	0.977 (0.361–2.64)	0.96
Chronic disease during pregnancy (no)	0.459 (0.099–2.127)	0.31	0.514 (0.12–2.199)	0.37
History of psychiatric disease (no)	3.924 (1.244–12.379)	0.02	2.692 (0.95–7.634)	0.06
Length of stay at home during the pandemic	1.004 (0.965–1.045)	0.83	1.013 (0.975–1.053)	0.52
Isolation precautions taken during the pandemic				
Use of mask	1.859 (0.416–8.316)	0.42	1.187 (0.277–5.084)	0.82
Use of mask and gloves	1.82 (0.322–10.271)	0.50	0.939 (0.171–5.153)	0.94
Concern about getting infected by the coronavirus (no)	6.064 (1.203–30.571)	0.03	0.947 (0.353–2.54)	0.91
Following Covid-19 news (no)	1.934 (0.304–12.295)	0.49	3.349 (0.337–33.256)	0.30
Presence at home of an individual older than 65 years of age (no)	2.319 (0.996–5.395)	0.05	1.614 (0.71–3.666)	0.25
Receiving regular antenatal care				
Yes	0.283 (0.109–0.734)	0.01	0.523 (0.2–1.367)	0.19
No	2.298 (0.225–23.448)	0.48	2.185 (0.328–14.553)	0.42

Abbreviations: 95%CI, 95% confidence interval; Covid-19, coronavirus disease 2019; BAI, Beck Anxiety Inventory; BDI, Beck Depression Inventory; OR, odds ratio.

Notes: *Cox & Snell R ^2^ : 0.231; Nagelkerke R ^2^ : 0.380; accuracy: 0.870. ** Cox & Snell R ^2^ : 0.112; Nagelkerke R ^2^ : 0.192; accuracy: 0.867; *p* < 0.05.

## Discussion

The present study aimed to determine the levels of anxiety and depression and related factors in Turkish pregnant women during the COVID-19 pandemic. Our findings showed that 53.9% of the pregnant women had anxiety and 54.3% of them had depression symptoms at varying degrees, from mild to highly severe. Lebel et al. (2020), 27 in a study with a similar sample, found a rate of anxiety of 59%, amd a rate of depression of 37%. In China, Wu et al. 28 found a prevalence of depression ofs 34.2%. In a study 21 in Iran, the prevalence of anxiety was determined as 43.9%. In a study 29 in India, the authors stated that the general prevalence of moderate and severe depression was of 13.2% ( *n* = 66), and the prevalence of moderate and severe anxiety disorder was of 9.8% ( *n* = 49). These studies have emphasized that the levels of anxiety and depression have increased among pregnant women in comparison to before the pandemic. While similar rates have been reported in a limited number of studies conducted in different countries during the COVID-19 pandemic, it was striking that the anxiety and depression rates in our study were a bit higher. 30 Examining the studies in the literature, one can see that they were conducted at the beginning of the pandemic (February-March 2020), while ours was conducted 6 months after the pandemic had started. The extension of the pandemic and the uncertain nature of this time may have increased the levels of anxiety and depression. At the same time, these findings emphasize the need for urgent interventions to increase the state of psychological wellbeing during pregnancy.

According to the results of the logistic regression analysis, the variables of the pregnant women (experiencing concern about their health, having an unplanned pregnancy, presenting history of psychiatric disease, and not attending regular antenatal follow-ups) were determined as predictors of anxiety symptoms. As a predictor of depression symptoms, only the status of being concerned about health was determined. During the pandemic, various nationwide precautions are being taken to reduce the spread of the virus, including social distancing, curfews, and self-isolation. At the same time, following the announcement of the start of the second wave of the pandemic worldwide, the numbers of infections and deaths continued to increase fast. Considering all these factors, it is inevitable for pregnant women, who are considered a risk group, to have concerns about their health. Similar to our study, the study by Lebel et al. (2020) 27 conducted on pregnant women found that the fact that pregnant women had concerns about their health and that of their babies increased their anxiety levels. In similar studies 17 31 32 33 conducted before the pandemic, history of psychiatric disease, not receiving regular antenatal care, and unplanned pregnancies were identified as risk factors affecting the prevalence of anxiety and depression. In their study on a Turkish population in the pandemic period, Özdin and Bayrak Özdin 34 determined that the female gender and history of psychiatric disease were risk factors for anxiety and depression. This is why it was an expected finding that, during the pandemic, pregnant women had relatively high levels of anxiety and depression.

In the present study, we determined that the regular practice of exercise and the presence of social support were not among the risk factors affecting the levels of anxiety and depression among the pregnant women. This finding contradicts with that of similar studies that have shown that physical activity and social support are associated with reduced anxiety and depression symptoms. 35 36 37 However, Lebel et al. 27 did not find a relationship between increased physical activity and reduced anxiety either. 22 The rate of regular physical activity in the pregnant women in our sample was of 36.1%. It may be stated that isolation measures taken during the pandemic (curfew, social distancing etc.) have led to a limitation in physical activity, especially in metropolitan areas, where the pandemic is intensely experienced. Encouraging pregnant women to perform physicala activities while taking the necessary protective measures is one of the most important interventions among the strategies to cope with the symptoms of anxiety and depression.

Social support is an important determinant of physical and psychological wellbeing, especially during pregnancy, when individuals are taking on new responsibilities and roles. Supportive social relationships affect mental health directly by encouraging positive health behaviors, increasing positive emotions, strengthening emotional regulation, and indirectly reducing reactions to physical stress. 38 In the present study, the prevalence of social support was of 75.5%. In the traditional structure of the Turkish society, the belief that pregnant and postpartum women should not be left alone is prevalent. In general, during pregnancy, support is provided by the mothers or grandmothers of the pregnant women, who, since they are generally older than 65 years of age, are also in another risk group for Covid-19. For this reason, during the pandemic, for the fact that pregnant women may have to provide care for family members who live in the same residence as them may comprise an additional risk factor. Moreover, unlike similar studies, the fact that the present study was conducted during the pandemic shows how strong the effect of the pandemic was on the psychological health of the pregnant women, even in the presence of social support.

One of the limitations of the present study was that its cross-sectional design may not completely reflect the causality in the relationship between the sociodemographic variables and the levels of anxiety and depression. Another limitation was that it was conducted in the province of Istanbul, which concentrated ∼ 60% of the cases of Covid-19 in Turkey. Therefore, the present study may not represent all pregnant women in Turkey. The small sample size is among the most important limitations of the study.

The Covid-19 pandemic was found to cause hemodynamic changes in the brain. 39 The present study mainly used self-reported questionnaires to measure psychiatric symptoms, and did not make clinical diagnoses. The gold standard to establish psychiatric diagnoses involves structured clinical interviews and functional neuroimaging. 40 41 42

## Conclusion

Anxiety and depression levels were present in about half of the pregnant women included in the present study. The risk factors to develop anxiety and depression were smoking, concerns about health and getting infected with the coronavirus, history of psychiatric disease, and sundergoing regular antenatal care. The present study also showed that pregnant women are vulnerable to changes in mental status during the pandemic, and special care should be taken to overcome the high levels of anxiety and depression brought about in this period of uncertainty and stress. Thus, we recommend that healthcare professionals perform psychosocial assessments in addition to physical examinations in antenatal follow-ups, and take precautions by determining the pregnant women under risk in the early period. Furthermore, health commissions should prepare national-level psychological crisis intervention guidelines. Vaccination is vital in the fight against Covid-19. We recommend the planning of a research on the perceptions of pregnant women against the Covid-19 vaccine and their willingness to get vaccinated.
